# Influence of radiant exposure values from two third generation LED curing units on polymerization profile and microhardness of orthodontic composite under ceramic and metallic brackets

**DOI:** 10.1590/2177-6709.26.1.e2119150.oar

**Published:** 2021-03-10

**Authors:** Andrés Fernando Montenegro ARANA, Barbara JUSTUS, Andrés DÁVILA-SÁNCHEZ, Michele de Oliveira SUGAHARA, Ulisses COELHO, Paulo Vitor FARAGO, Cesar ARRAIS

**Affiliations:** 1 Universidade Estadual de Ponta Grossa, Departamento de Odontologia (Ponta Grossa/PR, Brazil).; 2 Universidade Estadual de Ponta Grossa, Departamento de Ciências Farmacêuticas (Ponta Grossa/PR, Brazil).; 3 Universidad San Francisco de Quito USFQ, Escuela de Odontología (Quito, Ecuador).; 4 Universidade de Guarulhos, Departamento de Odontologia (Guarulhos/SP, Brazil).

**Keywords:** Curing lights, Hardness, Orthodontic bracket, Polymerization

## Abstract

**Introduction:**

Third generation of LED light curing units might be used in short exposure periods for orthodontic brackets bonding.

**Objective:**

This study evaluated the effect of the different radiant exposure (RE) values: Manufacturers’ instructions (MI), ½ MI, 1/4 MI and Turbo mode. Two third-generation LED curing units were used: VALO^®^ and Bluephase 20i^®^ . The degree of conversion (DC) and Vickers hardness (VHN) of an orthodontic composite (OC) (Transbond XT) under metallic (MB) or ceramic brackets (CB) were measured.

**Methods:**

OC was applied to the bracket base, which was then placed over an attenuated total reflectance (ATR) table coupled to an infrared light spectroscope, or to a glass surface for the VHN analysis. The specimens were light-cured and DC values were calculated. The VHN was obtained in a microhardness tester. The data were analyzed with 2-way ANOVA followed by Tukey’s *post-hoc* test (pre-set α=0.05). Linear regression analysis evaluated the relationship between RE values and dependent variables.

**Results:**

CB allowed higher DC and VHN values than MB (*p*< 0.001). No significant difference was noted among groups when CB were used. For MB, MI groups showed the highest DC and VHN values. A significant, but weak relationship was found between delivered RE values and dependent variables.

**Conclusions:**

The decrease in RE values from third generation LED CU did not jeopardize the DC values when CB were used, but can compromise DC and VHN values when MB are used.

## RESUMO

**Introdução:**

A terceira geração de LEDs fotopolimerizadores pode ser utilizada em curtos períodos de exposição para a colagem de braquetes ortodônticos.

**Objetivo:**

O presente estudo avaliou o efeito dos diferentes valores de irradiância (IR): instruções do fabricante (IF), ½ IF, ¼ IF e modo Turbo. Dois fotopolimerizadores LED de terceira geração (VALO^®^ e Bluephase20i^®^) foram utilizados. Foram mensurados o grau de conversão (GC) e a dureza Vickers (VHN) de um compósito ortodôntico (CO) (Transbond XT) sob braquetes metálicos (BM) ou cerâmicos (BC).

**Métodos:**

O compósito ortodôntico foi aplicado na base do braquete e foi posicionado sobre uma mesa de refletância total atenuada (ATR) acoplada a um espectroscópio de infravermelho ou a uma superfície de vidro para análise de VHN. As amostras foram fotopolimerizadas e os valores de GC foram calculados. O VHN foi obtido em um microdurômetro. Os dados foram analisados com ANOVA de 2 fatores seguida do teste *post-hoc* de Tukey (predefinido α = 0,05). A análise de regressão linear avaliou a relação entre os valores de IR e as variáveis dependentes.

**Resultados:**

BC permitiu valores maiores de GC e VHN do que BM (*p*<0,001). Nenhuma diferença significativa foi observada entre os grupos quando BC foi utilizado. Para BM, os grupos de IF mostraram os maiores valores de GC e VHN. Uma relação significativa, mas fraca, entre os valores de IR entregue e as variáveis dependentes foi encontrada.

**Conclusões:**

A diminuição dos valores de IR dos fotopolimerizadores LED de terceira geração não prejudicou os valores de GC quando BC foram utilizados, mas pode comprometer os valores de GC e VHN quando BM são utilizados.

## INTRODUCTION

The treatment success with fixed orthodontic appliance depends substantially on the accurate bracket bonding to enamel surface. The “adhesive” dentistry became viable after the introduction of the enamel etching with phosphoric acid, by Buonocore,^1^ and the release of resin composites. In Orthodontics, this advance allowed predictable direct bonding of brackets to enamel surface.^2^

Different types of orthodontic composites (OC) have been used in clinical practice. Some are light-cured materials, others are self-cured resins, and there are also dual-cured OCs, which have both photoinitiators and self-curing components in their composition.^3^ Although these products have shown acceptable mechanical proprieties,^4^ the use of light-curing units (LCU) is required regardless of the OC type, to ensure that brackets are bonded without wasting chairtime, once photo-activated polymerization is considerably faster than self-cured polymerization.^5^ ^,^ ^6^ Indeed, in order to provide optimal degree of conversion (DC) and mechanical properties of OCs, the radiant emittance values must be considerably high.^7^ In this regard, recently, third generation light-emitting diode (LEDs) curing units have become available for dental practitioners.^8^ Also known as multi-peak LCUs, these LED devices are capable of emitting light with varying wavelength ranging from 390 nm to 490 nm.^8^ ^,^ ^9^

The polymerization efficiency of OCs depends on the radiant emittance values, exposure time, and the light source. In general, the physical and mechanical properties of resin-based materials are closely related to the DC.^10^ ^,^ ^11^ In addition, poor monomer conversion results in monomer leaching and the release of plasticizers and polymerization initiators.^12^ ^,^ ^13^ Such an issue is a matter of concern as monomer leaching from poorly polymerized resin-based composites has been associated with metabolic diseases, problems in gene expression,^14^ and also problems in immune responses.^15^

Despite the advances in adhesion and LED technology, the currently used bonding protocol for metallic and ceramic brackets still remains a time-consuming procedure, once clinicians usually avoid short exposure intervals. Longer chairtime also increases the chance of bonding failures due to contamination, mainly in posterior and lower teeth.^16^ In this regard, some *in vitro* studies have evaluated the influence of LCU types and shorter exposure periods of LCU on monomer conversion of OCs.^17^ Although most studies properly addressed this issue and observed the influence of exposure period and LCU type, the differences in products and curing protocols among studies resulted in controversial findings. In addition, none of these studies evaluated the influence of both metallic and ceramic brackets interposed between the LCU tip and the OC layer on DC values and kinetics of polymerization. To date, no information is available in the literature regarding the use of powerful third generation LED CUs at short exposure periods on OC polymerization. 

Thus, this study evaluated the effects of varying radiant exposure (RE) values comprising short exposure intervals to light emitted from two high power LED CUs on DC, maximum rate of polymerization (Rp_max_) and Vickers hardness (VHN) of one commercially available OC in a clinical simulated bonding procedure of metallic or ceramic brackets. The research hypotheses were: (1) the delivery of lower RE values decreases DC, Rp_max_, and VHN values of OC layers after exposure to light emitted from polywave LED CU through either metallic or ceramic brackets; (2) there is direct and positive relationship between RE values and DC or VHN values; and (3) the DC, Rp_max_, and VHN values after LED exposure through ceramic brackets are higher than those observed after exposure through metallic brackets. 

## MATERIAL AND METHODS

### ANALYSIS OF THE DEGREE OF CONVERSION

A commercially available OC (Transbond™ XT, 3M, California, USA) was used in the present research. The metallic and ceramic brackets (Roth prescription, Morelli, Sorocaba/SP, Brazil) were employed for the tests. The third generation LED CUs (VALO^®^, Ultradent Products Inc, South Jordan, UT, USA; and Bluephase 20i^®^, Ivoclar Vivadent Inc., Ivoclar Vivadent, Schaan, Liechtenstein) were evaluated. Radiant emittance values of light emitted by the LED CUs were measured with a portable laser power meter (407A, Newport Corporation, CA, USA). In order to simulate a clinical situation, the OC was applied to the orthodontic bracket according to the manufacturer’s instructions. The orthodontic bracket containing the OC layer was placed on the diamond surface of an attenuated total reflectance (ATR) table (Satandard Golden Gate, Specac, Woodstock, GA, USA) coupled to an infrared light spectroscope (FTIR, Tensor 27, Bruker Optik GmbH, Ettlingen, Germany), so the OC layer was in contact with both ATR diamond surface and the bracket base. The LCU tip was placed against the bracket and the specimens were exposed to light emitted either from VALO or Bluephase20i at the following exposure intervals and exposure modes: Manufacturers’ instructions (MI), half MI, one quarter MI and Turbo mode, in which the RE values were delivered at shorter exposure periods and higher radiant emittance than that of MI or half MI, as shown in Table 1. Therefore, the RE values delivered to the specimens ranged from 6 J/cm^2^ to 22.9 J/cm^2^ when metallic brackets were used, and from 2.85 J/cm^2^ to 11.4 J/cm^2^ when ceramic brackets were used (Table 1). In addition, in an attempt to simulate the clinical scenario where LCU tip is placed on the mesial and distal portion of the metallic bracket, the LCU tip was placed in two directions, so light was delivered for half exposure period in each side. Conversely, when ceramic brackets were used, the LCU tip was placed directly against the ceramic bracket. Seven specimens were evaluated for each experimental group (n=7). 

**Table 1:** Experimental groups evaluated in the study.

LED LCU	Bracket	Exposure mode	Radiant emittance (mW/cm^2^)	Exposure period (s)	Radiant Exposure values (J/cm^2^)	Group name	Description
Valo	Metallic	Standard power	1273	18	22.9	MI	Control
				9	11.5	½ MI	Half control time
				5	6.4	¼ MI	Quarter control time
		Plasma Emulation	3200	6.6	21.1	Turbo	Plasma
	Ceramic	Standard power	1273	9	11.5	MI	Control
				4.5	5.7	½ MI	Half control time
				2.25	2.9	¼ MI	Quarter control time
		Plasma Emulation	3200	3.33	10.7	Turbo	Plasma
Bluephase	Metallic	Standard power	1136	20	22.7	MI	Control
				10	11.36	½ MI	Half control time
				5	5.68	¼ MI	Quarter control time
		Turbo	2045	6	12.3	Turbo	Turbo
	Ceramic	Standard power	1136	10	11.36	MI	Control
				5	5.68	½ MI	Half control time
				2.5	2.84	¼ MI	Quarter control time
		Turbo	2045	3	6.12	Turbo	Turbo

Infrared spectra were collected between 1680 and 1500 cm^−1^ at a rate of one spectrum per second (16 scans/spectrum) at 4 cm^−1^ resolution. Data were collected from the moment the infrared scan demonstrated that the resin was stabilized on the ATR surface and the bracket had been placed. Spectra were recorded continuously during each 1-second interval for 10 minutes.

DC values were calculated using standard methods that evaluated changes in the ratios of aliphatic-to-aromatic C=C absorption peaks (1636 cm^−1^/1608 cm^−1^) in the uncured and cured states obtained from the infrared spectra.^18^ Rp_max_ values corresponded to the highest rate of polymerization (percentage) and were calculated based on the differences between DC values measured in sequential, 1-second intervals throughout the 10-min analysis of each specimen.

DC equation:

$$DC\equiv 1-\frac{[\;abs\;(C=C\;alifatic\;)/abs(C\ldots C\;aromatic\;)]\;polymer\;}{[\;abs\;(C=C\;alifatic)/abs\;(C\ldots \;Caromatic\;)]\;monomer\;}\times 100$$

DC: Degree Conversion (%)

abs: absorbance

### MICROHARDNESS TEST

For VHN, 80 metallic and ceramic brackets were used. Prior to OC placement, both brackets had the rough back-surface smoothed to remove any retention, so the OC could be removed and the bracket could be reused. The OC was applied to the bracket according to MI, and the set orthodontic bracket/composite layer was placed on a glass surface. The LCU tip was placed on the bracket and the specimens were exposed to the LED CUs at varying exposure intervals, as previously describe.

**Figure 1:**
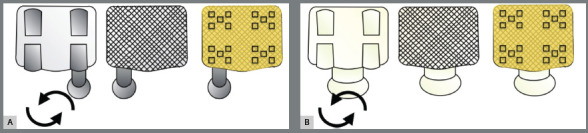
Illustrative image of OC layer on metallic ( A ) and ceramic ( B ) brackets and all indentations made in each corner.

Hardness was immediately evaluated with microhardness indenter (Microhardness tester- Shimadzu Corporation, Kyoto, Japan). VHN analysis was performed as reported by Garcia-Contreras et al.^19^ A diamond indenter was applied to the OC surface at 5 N or 50 Kgf, and a 15-s indentation interval was used. Five indentations were obtained on each corner, resulting in a total of 20 indentations in each specimen (Fig 1). 

## STATISTICAL ANALYSIS

Because light beam profiles of the evaluated LCUs were not similar, no comparison between results of each tested LCU was made. Therefore, the DC, VHN and Rpmax (%/s) values were evaluated using 2-way ANOVA (“exposure mode” and “bracket type” as independent variables) within each LCU, followed by Tukey’s *post-hoc* test at a pre-set alpha of 5%. Linear regression analysis was performed to evaluate the relationship between RE values and either DC or VHN values. ***Post-hoc*** power analysis was performed for the statistical analyses of DC, Rp_max_, and VHN values. All statistical analyses were performed using statistical softwares (Prism for Macintosh version 6.0, GraphPad Software Inc., CA, USA, and Statistics 19, SPSS Inc, IBM Company). 

## RESULTS

### DEGREE OF CONVERSION (DC) AND RP_MAX_ VALUES

The DC (%) and Rp_max_ (%/s) values after exposure to VALO or Bluephase20i at varying RE values (J/cm^2^) are shown in Tables 2 and 3, respectively. Two-way ANOVA detected statistical significance for the independent variable “exposure mode” and “bracket type” (*p<*0.001), as well as for the statistical interaction between the independent variables (*p<*0.001), regardless of LCU type, for both DC and Rp_max_ values. 

**Table 2:** Mean (SD) DC values after exposure to light emitted from LCUs at varying radiant exposure values under metallic and ceramic brackets.

	Irradiation	Metallic	Ceramic
VALO	MI	35.8 (3.4)^Ab^	47.0 (1.6)^Aa^
	½ MI	27.0 (4.4)^BCb^	44.7 (1.4)^Aa^
	¼ MI	21.6 (6.9)^Cb^	43.0 (1.9)^Aa^
	Turbo MI	31.6 (5.4) ^ABb^	44.4 (1.5)^Aa^
Bluephase20i	MI	35.0 (3.5) ^Ab^	45.6 (1.4)^Aa^
	½ MI	28.0 (3.1)^Bb^	43.6 (1.2)^Aa^
	¼ MI	23.7 (6.1)^Bb^	44.0 (2.3)^Aa^
	Turbo MI	28.2 (5.8)^Bb^	46.2 (2.0)^Aa^

* Significant differences between means are followed by different superscript letters (uppercase within column; lower case within row, pre-set alpha of 0.05). No comparison between results of different LCUs was performed.

**Table 3:** Mean (SD) Rpmax values after exposure to light emitted from VALO and Bluephase 20i at varying radiant exposure values under metallic and ceramic brackets.

	Irradiation	Metallic	Ceramic
VALO	MI	2.7 (1.1)^Ab^	10.9 (1.5)^Ba^
	½ MI	2.4 (0.8)^Ab^	10.6 (1.0)^Ba^
	¼ MI	2.8 (1.0)^Ab^	11.0 (1.3)^Ba^
	Turbo MI	3.9 (1.8)^Ab^	14.0 (1.5)^Aa^
Bluephase20i	MI	2.6 (1.1)^Ab^	10.5 (1.6)^Ba^
	½ MI	2.4 (0.8)^Ab^	10.3 (1.2)^Ba^
	¼ MI	3.1 (1.2)^Ab^	11.2 (1.3)^Ba^
	Turbo MI	2.9 (0.8)^Ab^	14.0 (1.5)^Aa^

* Significant differences between means are followed by different superscript letters (uppercase within column; lower case within row, pre-set alpha of 0.05). No comparison between results of different LCUs was performed

Overall, the use of ceramic bracket resulted in higher DC and Rp_max_ values than the use of metallic bracket either when VALO or Bluephase20i were used. For both LCUs, no significant difference was noted among groups when ceramic brackets were used. However, within the group comprising the use of metallic brackets after exposure to VALO, the MI and Turbo groups showed the highest DC values. The DC values observed in 1/2-MI group were significantly lower than those of MI group (*p<*0.001) but not significantly different from those of Turbo group. The 1/4-MI group showed the lowest DC values (*p<*0.001), which were not significantly lower than those observed in 1/2-MI group. Within the groups comprising the use of metallic brackets after exposure to Bluephase20i, the MI group showed the highest DC values, which were significantly higher than those observed in the other groups (*p<*0.001). No significant difference in DC values was noted among the other groups. 

When orthodontic composite was exposed to light emitted from VALO or Bluephase20i placed over ceramic brackets, Turbo groups exhibited the highest Rp_max_ values (***p<*** 0.001). No significant difference was observed among the other groups, all of which showed significantly lower Rp_max_ values than did the Turbo groups (***p<*** 0.001). When metallic brackets were used, no significant difference in the Rp_max_ values was noted among groups, regardless of LCU type. 

**Figure 2:**
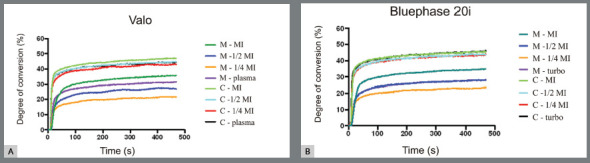
Representative real-time kinetic profile of monomer conversion of OC layer at varying exposure conditions under either metallic ( M ) or ceramic ( C ) brackets, during exposure to light emitted from VALO or Bluephase20i.

Figure 2 shows representative real-time profiles of kinetics of polymerization during exposure to light emitted from either VALO or Bluephase20i, when metallic or ceramic brackets were used. When ceramic brackets were used, similar real-time profiles were observed for all exposure modes, regardless of LED CU. Fast rise in DC values were noted during exposure to LCU light, then the rate of monomer conversion decreased when DC values reached approximately 38% to 40%, corresponding to polymer vitrification. Slow increase in DC values was noted after that period. 

Conversely, when metallic brackets were used, real-time profile of kinetics of polymerization was clearly affected by the exposure mode, regardless of LED CU. More specifically, the fast rise in monomer conversion was shorter when shorter exposure modes were used, in comparison to those observed for longer exposure modes. Therefore, when shorter exposure modes were used, the rate of monomer conversion slowed down at apparently lower DC values (ranging from approximately 15% to 25%) than those observed when longer exposure modes were delivered to the orthodontic composite. As a consequence, lower 10-min DC was noted after shorter exposure modes. The exception was noted when shorter exposures at high intensity were used (Plasma mode in VALO and Turbo mode in BLuephase20i). Despite the short exposure periods in those exposure modes, the resulting real-time profile of kinetics of polymerization was close to that observed when the orthodontic composite was exposed to LCU light following MI. 

### VICKERS MICROHARDNESS

The VHN values (Vickers) and standard deviation (SD) after exposure to VALO or Bluephase20i at varying RE values (J/cm^2^) are shown in Table 4. Two-way ANOVA detected statistical significance for the interaction between independent variables “exposure mode” and “bracket type”, regardless of LCU (*p<*0.001). 

**Table 4:** Mean (SD) VHN values after exposure to light emitted from the LCUs at varying radiant exposure values under metallic and ceramic brackets.

	Irradiation	Metallic	Ceramic
VALO	MI	42.4 (1.4)^Aa^	41.8 (2.1)^Aa^
	½ MI	32.7 (1.6)^Bb^	37.3 (2.2)^Ba^
	¼ MI	21.1 (1.0)^Cb^	30.0 (1.0)^Ca^
	Turbo MI	31.3 (1.8)^Bb^	35.7 (0.6)^Ba^
Bluephase20i	MI	40.1 (1.4)^Aa^	41.4 (1.1)^Aa^
	½ MI	29.7 (1.4)^Cb^	39.0 (1.3)^Ba^
	¼ MI	19.3 (1.5)^Db^	30.4 (1.4)^Da^
	Turbo MI	32.1 (1.3)^Bb^	36.1 (1.5)^Ca^

* Significant differences between means are followed by different superscript letters (uppercase within column; lower case within row, pre-set alpha of 0.05). No comparison between results of different LCUs was performed.

When the OC layer having metallic bracket was exposed to light emitted from VALO, MI group exhibited the highest VHN values, which were significantly higher than those of the other groups (*p<*0.001). No significant difference in VHN values was observed between 1/2-MI and Turbo groups, which in turn showed higher VHN values than did 1/4-MI group (*p<*0.001). The exposure to curing light through ceramic brackets resulted in significantly higher VHN values than the exposure through metallic brackets in most groups (*p<*0.001). The only exception was observed in MI groups, where no significant difference was noted between those groups having metallic brackets and those having ceramic brackets. 

When Bluephase20i was used, the exposure following MI instructions resulted in the highest VHN values, regardless of the bracket type. When the metallic brackets were used, Turbo MI groups promoted higher VHN values than did 1/2-MI (*p<*0.001), which in turn exhibited significantly higher VHN values than did 1/4-MI group (*p<*0.001). Similarly to the results obtained with VALO, exposure to light from Bluephase20i through ceramic bracket promoted higher VHN values than the exposure through metallic bracket in most groups, with the exception of MI group, in which no significant difference was observed in VHN values when ceramic brackets were used, in comparison to the values observed when metallic brackets were used.

### LINEAR REGRESSION ANALYSIS

**Figure 3:**
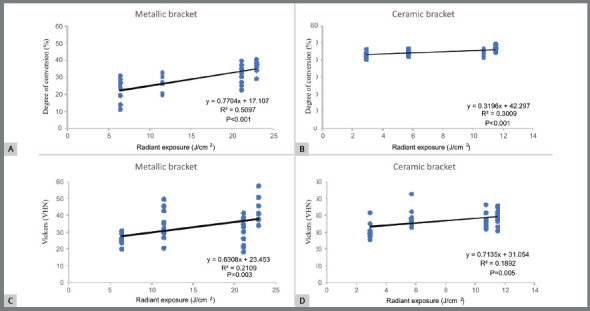
Regression analysis plot of DC and VHN values vs delivered RE by VALO through metallic (**A** and **C**, respectively) or ceramic brackets (**B** and **D**, respectively).

**Figure 4:**
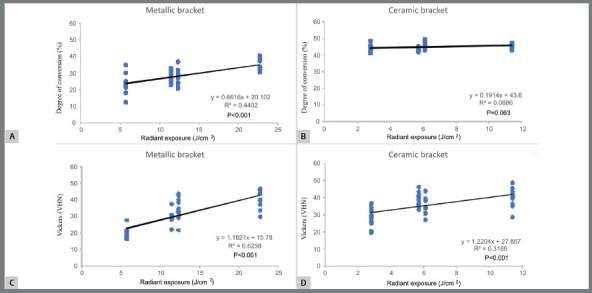
Regression analysis plot of DC and VHN values vs delivered RE by Bluephase20i through metallic (**A** and **C**, respectively) or ceramic brackets (**B** and **D**, respectively).

Figures 3 and 4 show the results of linear regression analysis of the relationship between RE values and both DC and VHN values, respectively. When metallic brackets were used, a significant, weak positive relationship was observed between applied RE values and either DC or VHN values after exposure to light emitted from VALO (*r* ^*2*^ = 0.510; *p*<0.001 for DC values; *r* ^*2*^ = 0.210; *p*=0.003 for VHN values) and Bluephase20i (*r* ^*2*^ = 0.440; *P*<0.001 for DC values; *r* ^*2*^ =0.626; *p*<0.001 for VHN values). Weaker relationship between RE values and DC or VHN values was observed when ceramic brackets were used, either when light was emitted from VALO (*r* ^*2*^=0.283; *p*<0.001 for DC values; *r* ^*2*^ =0.189; *p*<0.005) or when light was emitted from Bluephase20i regarding VHN values (*r* ^*2*^=0.317; *p*<0.001). The relationship between RE values and DC values was not statistically significant when Bluephase20i was used (*p=*0.063). 

## DISCUSSION

In the current study, the effects of varying RE on DC and VHN values of OC layer was influenced by the bracket type. More specifically, although no significant difference in Rp_max_ values was observed, most groups showed lower DC and VHN values when the delivered RE values corresponded to 1/2 and 1/4 of the MI, in comparison to the values observed in the control group when metallic brackets were used. Conversely, varying the RE values caused no significant difference in the DC and Rp_max_ values when ceramic brackets were used. Therefore, the first null hypothesis was partially accepted for the DC values and accepted for VHN values. These results are in agreement with previous findings,^20^ ^,^ ^21^ and demonstrated that the use of metallic brackets require longer exposure periods due to the detrimental effects of light attenuation caused by the presence of those brackets interposed between the OC layer and LCU tip. 

Although the reduction in RE values to 1/2 or 1/4 of that recommended in the MI caused lower VHN values in most experimental conditions, only a weak, linear, positive, significant relationship between RE values and either DC or VHN values was observed in most conditions. Indeed, no significant relationship was noted between RE values and the DC or VHN values when OC was exposed to light emitted from Bluephase20i through ceramic brackets (Figs 3 and 4). Therefore, the second null hypothesis stating that there is a direct relationship between RE values and either DC or VHN values was partly accepted. Such a weak relationship may be explained by the influence of the brackets interposed between OC and the LCU tip. When ceramic brackets are used, lower attenuation of the light emitted from LED LCUs is expected, in comparison to that when metallic brackets are used. As a consequence, even the lowest delivered RE values were capable of promoting close VHN values to or as high DC values as those obtained after following MI. 

Differently from the results observed when ceramic brackets were used, DC and VHN values of OC layers under metallic brackets were apparently more severely affected by the reduction in the delivered RE values, as previously reported in other study.^20^ This result may be attributed to the fact that curing light is entirely blocked by the presence of metallic bracket, so OC polymerization relied solely on the effects of light reaching the edge of metallic bracket. For this reason, higher RE values are required to ensure optimal polymerization and mechanical properties. As a consequence, apparently higher relationship between RE values and DC or VHN values was noted when metallic brackets were used, in comparison to that observed when ceramic brackets were used. Therefore, the second research hypothesis was accepted when metallic brackets were evaluated.

The lower attenuation in curing light caused by the presence of ceramic brackets, in comparison to that observed when metallic brackets are used, also helps explaining the higher Rp_max_ values and the consequent higher DC and VHN values observed when ceramic brackets are used. Therefore, the third hypothesis was accepted for DC, Rp_max_ and VHN values. This finding corroborates previous evidence that Rp_max_ values are related to radiant emittance values rather than to the exposure interval or RE values^6^ ^,^ ^22^ ^,^ ^23^ and also helps explaining why the use of Plasma mode in VALO and Turbo mode in Bluephase20i resulted in higher Rp_max_ values than those observed in the other groups when ceramic brackets were used, despite the shorter exposure interval. As a consequence, the DC and VHN values after exposure to shorter exposure interval such as those applied when Plasma (VALO) or Turbo (Bluephase20i) were as high as those observed in the control groups (manufacturers’ instructions) in most experimental conditions. In addition, the profile of polymerization kinetics in groups exposed to Plasma or Turbo modes at short exposure periods were similar to those observed in the control groups, corroborating the exposure reciprocity law previously observed in most photo-activated resin-based composites.^24^ ^,^ ^25^

The decrease in VHN values as a result of the reduced RE values delivered to OC layer were not closely related to that observed in the DC values. For instance, the delivery of 1/2 and 1/4 of the MI’s recommended RE values though ceramic brackets decreased DC values in 4.9% and 8.5% in comparison to the values observed after exposure following MI when VALO was used, respectively. Conversely, the same exposure modes through ceramic brackets decreased VHN values in approximately 10.8% and 28.2% in comparison to the values after exposure according to MI. These results contradict the well documented correlation between monomer conversion and hardness of resin composites.^26^ Such a lack of correlation between the DC and VHN values may be attributed to the difference between the regions of the OC surface where DC and VHN analyses were performed. More specifically, DC analysis was performed in the middle of the OC layer, while VHN analysis was performed at the corners of the OC layer. Because of the distribution of LED chips, the light emitted by most multi-peak, third generation LED CUs is not uniformly distributed regarding the irradiance and wavelength on the irradiated surface.^27^ As a consequence, it is possible that lower radiant emittance values were delivered at the corners in comparison to those reaching the middle of the OC layer. 

In this study, DC and VHN values were measured approximately 7 min after light exposure to LED CUs. Therefore, once polymerization of resin-based composites may continue for over 24 hours, further increase in DC values is expected. However, it should be emphasized that evaluating initial monomer conversion and hardness of orthodontic composites is crucial for the success of orthodontic treatment, as these products are subjected to tension soon after they are exposed to curing light. Thus, OCs should achieve optimal monomer conversion and mechanical properties within the first minutes after exposure to light emitted from LED CUs.^25^ In addition, the current results were based on one commercially available photo-activated composite with camphorquinone as the main photoinitiator. As a consequence, the results should not be extrapolated to products with other photoinitiators. The current results cannot predict the actual influence of these exposure modes on bond strength and long-term consequences of bonding to enamel surface. Further investigation is required to address these issues. 

## CONCLUSION

Based on the current findings and within the limitations of the present study, it was possible to conclude that:

» Despite the slight decrease in VHN values, the decrease in RE values by the reduction in exposure interval did not jeopardize the DC or RPmax values when ceramic brackets are used, while DC and VHN values may be compromised by the reduction in the exposure interval when metallic brackets are used.
» A significant, but weak relationship was noted between RE values and DC and VHN values, regardless of bracket type.
» Exposure of OC to light through ceramic brackets results in higher DC, Rp_max_, and VHN values than exposure through metallic brackets.
